# Editorial: Out-of-field second primary cancer induction: Dosimetry and modelling

**DOI:** 10.3389/fonc.2022.1076792

**Published:** 2022-12-05

**Authors:** Beatriz Sánchez-Nieto, Liliana Stolarczyk, Alexandru Dasu, Wayne D. Newhauser, Francisco Sánchez-Doblado

**Affiliations:** ^1^ Institute of Physics, Faculty of Physics, Pontifical Catholic University of Chile, Santiago, Chile; ^2^ Danish Center for Particle Therapy, Aarhus University Hospital, Aarhus, Denmark; ^3^ The Henryk Niewodniczański Institute of Nuclear Physics, Polish Academy of Sciences, Krakow, Poland; ^4^ Medical Radiation Sciences, Department of Immunology, Genetics and Pathology, Uppsala University, Uppsala, Sweden; ^5^ The Skandion Clinic, Uppsala, Sweden; ^6^ Department of Physics and Astronomy, Louisiana State University, Baton Rouge, LA, United States; ^7^ Faculty of Medicine, University of Seville, Seville, Spain

**Keywords:** peripheral dose, out-of-field dose, photon radiotherapy, proton radiotherapy, radiation induced cancer, second cancer

Second primary cancer induction is a growing concern, particularly for the younger cancer patient population with a longer life expectancy, as demonstrated by the increasing number of publications on the topic. Still, there is much work to do (1), such as assessing problems associated with the dosimetry under no reference conditions (particularly in proton treatments) or the presence of mixed-fields. Additionally, due to the poor performance of commercial treatment planning systems (TPS) in stray dose calculations for photon (2) and proton radiotherapy (RT), the development and implementation of computational tools are needed for out-of-field dose estimation in a systematic way. Thus, dosimetric information might be part of databases for cancer patients treated with modern RT techniques together with detrimental outcomes such as second primary cancers. The latter will improve existing risk models, which should also be considered during RT plan optimization.

This issue focuses mainly on the dosimetric and modeling aspects of the out-of-field radiation generated during photon (Sa et al., Saint-Hubert et al., Sánchez-Nieto et al., Vogel et al.), proton (Eliasson et al., Carles Domingo et al., Hoey et al., Mares et al. and Saint-Hubert et al.) therapies as well as a comparison between the second therapies (Knežević et al.). A review (Romero-Expósito et al.) of the current status of the problems encountered when determining out-of-field doses in proton therapy in young patients is also part of this issue.

Sa et al. study out-of-field doses during photon RT of a brain tumor in a pediatric phantom using TLD measurements and Monte Carlo (MC) simulations for three-dimensional conformal radiotherapy (3DCRT), and intensity modulated radiotherapy (IMRT). Similarly, Vogel et al. present a planning exercise on breast irradiation comparing the two mentioned techniques with the addition of either sequential or simultaneous integrated external RT or interstitial multicatheter brachytherapy. The results from both papers show that in terms of peripheral doses, the 3DCRT, combined with an interstitial multicatheter brachytherapy boost, is the most suitable technique. However, it is essential to highlight the apparent advantages of IMRT (e.g., better target conformality and thus lower NTCP) when considering a more comprehensive biological index performance (3).

Two studies delve into developing computational tools for stray dose calculation in photon RT. Saint-Hubert et al., present the experimental validation of Hauri’s model (4) and a fast Monte Carlo algorithm, both coded as a script running in the Eclipse Treatment Planning System (TPS) (v. 15.6). Discrepancies between the analytical model and MC were in general smaller than 40% and 20%, respectively. Sánchez-Nieto et al. propose a relatively simple analytical model which, from minimum information of the associated RT plan, calculates the DVH of out-of-field organs through a graphical user interface (termed Periphocal 3D). The model was trained using 3D dose volume data calculated by MC simulations and allows peripheral dose calculation for isocentric 3DCRT, IMRT, or VMAT with an uncertainty of ±23%. Comparison of the model with TLD measurements inside an anthropomorphic phantom for a VMAT treatment and with a previously published physics-based analytical model (5) showed agreement within the model’s uncertainties. These two implementations of out-of-field dose computational tools ease the theoretical second cancer risk assessment, proper analysis of data derived from epidemiological reports, and treatment plan optimization, considering second primary cancer probabilities as an objective function.

The second part of this issue deals with out-of-field doses from proton irradiation. Compared to conventional photon therapy, proton therapy (PT) has the potential to reduce exposure and radiation risks outside the target volume. Nevertheless, there is still a concern that stray radiation can increase secondary cancer risks (particularly in young patients who are more radiosensitive). As mentioned in the review (Romero-Expósito et al.), most of the published research has been conducted for passive scattering installations, while studies on the more recent scanning proton beams dominate this issue. Hoey et al., Mares et al. and Eliasson et al. analyze the complex dependences of patient and proton field size, range, modulation width, or the use of a range shifter on the peripheral dose. Hoey et al. present a general MC model as the first step toward a tool for predicting out-of-field neutron doses in scanning proton therapy facilities. Simulations with the verified model enabled a detailed study of the neutron ambient dose equivalent H*(10) variation with plan parameters. They concluded that it is not enough to normalize the out-of-field neutron doses only to the target dose, as done in most of the published papers, but that it is essential to provide additional properties of the treatment plan, such as range, modulation, and field size. Mares et al. show the impact of the (pediatric) patient size on H*(10) with a focus on the possibility that parents or other comforters can remain inside the treatment room during scanning PT (which may be beneficial when it is not possible to treat children under anesthesia). However, it is acknowledged that further work considering other factors such as field size, range, modulation width, or the presence and position of the range shifter is required before general recommendations can be given. In Eliasson et al., the influence of beam energy, detector and range-shifter positions on the absorbed dose, LET, and dose equivalent was investigated using MC simulations and experimental measurements with microdosimetric tissue-equivalent proportional counters (TEPCs). They showed that the proton contribution scattered directly from the range shifter dominates in some situations, and although the LET of the radiation is decreased, H*(10) is increased by a factor of up to 3.

The complex and different dependencies of proton technique, patient size, and treatment parameters on the stray dose distribution may make non-trivial the development of methodologies for the estimation of out-of-field dose equivalent. This is the aim of the works by Saint-Hubert et al. and Domingo et al. The first study presents the evaluation of the accuracy of a computational method (based on the TOPAS framework) compared to experimental measurements. The development of such an MC framework could lead to tools for dose optimization in pediatric PT. A different approach is followed by Domingo et al. who propose a reproducible methodology for head and abdomen PT treatments (based on measurements of photon and neutron fluences using passive dosimeters inside an anthropomorphic phantom and complemented by the MC generation of the neutron spectra at the same points) that allows calculation of the dose equivalent to out-of-field organs in passive facilities.

As a finishing touch, the work by Knežević et al. analyzes and compares out-of-field neutron and non-neutron organ doses inside 5- and 10-year-old pediatric anthropomorphic phantoms from PT for a brain tumor. Out-of-field doses measured using intensity-modulated proton therapy (IMPT) were compared with IMRT, 3DCDRT, and Gamma Knife radiosurgery. The total organ dose equivalent expressed as the sum of neutron and non-neutron components in IMPT was found to be significantly lower (2-3 orders of magnitude) compared with photon RT techniques for the same target dose.

## Author contributions

BS-N drafted the editorial, and AD, FS-D, LS, and WN contributed by summarizing the published manuscripts for which they acted as editors, revised the draft, and make suggestions for the final text. All authors contributed to the article and approved the submitted version.
